# JYYS Granule Mitigates Renal Injury in Clinic and in Spontaneously Hypertensive Rats by Inhibiting NF-*κ*B Signaling-Mediated Microinflammation

**DOI:** 10.1155/2018/8472963

**Published:** 2018-11-26

**Authors:** Dong Yan, Bowen Yue, Muyan Qian, Lili Zhao, Zihan Zhang, Hui Qian, Shihai Yan, Yuliang Qian, Zhuyuan Fang

**Affiliations:** ^1^Jiangsu Province Hospital of TCM, Affiliated Hospital of Nanjing University of TCM, Nanjing, China; ^2^Nanjing University of TCM, Nanjing, China; ^3^Nanjing Foreign Language School, Nanjing, China

## Abstract

**Introduction:**

Hypertensive renal damage is a chronic and life-threatening kidney disease all over the world. The traditional Chinese medicine Jiang Ya Yi Shen (JYYS) granule has been a perfect drug for patients with hypertensive renal injury in clinic for 20 years in China. However, the molecular mechanism of JYYS granule remains unknown in treatment of this disease.

**Methods:**

The clinic data were from this study's patients. The clinical symptoms of patients were indicated by (N-Acetyl-*β*-D-Glucosaminidase) NAG, (albumin) Alb, and (*β*2-microglobin) *β*2-MG content in urinary of patients, and renal artery's hemodynamic parameters including (pulse index) PI, mean velocity of the arterial blood (Vm), minimum velocity of the diastolic stage (Vdmin) and peak velocity of the systolic wave (Vsmax). To further observe the effect of JYYS granule on renal damage, the rats were included in six groups: normal rats (WKY), spontaneously hypertensive rats (SHR), positive drug-treated rats (Benazepril), low dose JYYS (L), middle dose JYYS (M), and high dose JYYS (H). Then, we observed the effect of JYYS on renal function, renal tubules, inflammatory cell infiltration, and small artery thickening, and we explored the potential mechanism of JYYS in treatment of renal injury.

**Results:**

JYYS significantly improved the clinic symptoms of patients with hypertensive nephropathy by downregulating NAG, Alb, and *β*2-MG content in urinary of patients and by decreasing renal artery's hemodynamic parameters including PI, Vm, Vdmin, and Vsmax. In SHR, JYYS significantly improved renal function including creatinine clearance rate, urinary albumin/creatinine, *β*2-MG/creatinine and arteria caudalis pressure in SHR. Secondly, light and electron microscopic examinations told that after administration of JYYS and Benazepril, the mesangial region exhibited no hyperplasia and renal capsule did not expanded, and there no abnormalities were observed in renal tubules, inflammatory cell infiltration and small artery thickening in SHR. Thirdly, JYYS exhibited its protective role by inhibiting nuclear factor kappa beta signaling-mediated micro-inflammation cytokines including interleukin 6 (IL-6), tumor necrosis factor *α* (TNF-*α*), intercellular cell adhesion molecule-1 (ICAM-1), and monocyte chemotactic protein 1 (MCP-1) in SHR.

**Conclusion:**

JYYS is a promising prescription of Chinese medicine for patients with hypertension and hypertensive renal damage.

## 1. Introduction

Chronic kidney disease (CKD) has been a significant health problem all over the world. It is a life-threatening disease frequently associated with hypertension, progression to renal fibrosis, and eventual renal failure [1, 2]. Prevention of CKD may improve renal damage and cardiovascular disease [2]. The therapeutic outcome of CKD remains unsatisfactory owing to the unclear understanding of pathological mechanisms [3]. Hypertension is a leading cause of kidney diseases by modulating interstitial fibrosis, glomerulosis, and mesangial cell growth. High blood pressure could raise hyperfiltration resulting in increase of proteinuria, activation of inflammatory signaling, and development of renal fibrosis [4–8]. Microinflammatory cytokines are expressed much more in patients with hypertension than in healthy people, suggesting that these cytokines were involved in the process of hypertensive renal damage [9–12].

Nuclear factor-*κ*B (NF-*κ*B) performs an essential role in the formation of hypertensive nephropathy [13, 14]. NF-*κ*B activity can be inhibited by I*κ*B. The inhibitor protein I*κ*B could be phosphorylated by a variety of stimuli and followed by its ubiquitination and subsequent degradation. In the end, NF-*κ*B migrates to the nucleus and promotes transcription of its targets, including IL-6, TNF-*α*, ICAM-1, and MCP-1 [13, 14]. The above inflammation-related genes often trigger renal inflammation [13, 14]. The SHR has been used widely for an animal model of human essential hypertension [15–18]. This animal model always develops interstitial fibrosis and glomerular sclerosis accompanied by renal inflammatory infiltration that gives birth to renal injury [15–18].

Ancient traditional Chinese medicine (TCM) has been practiced for over 2,000 years [19]. Modern Western medicine was introduced in the 19th century. Both Western medicine and Chinese medicine are prepared for management of hypertension and renal failure [20–23]. As a traditional Chinese medicine, JYYS granule is the patented prescription prepared by Jiangsu Province Hospital of TCM and it has been used in clinic to cure hypertension-induced asthenic yin causing predominant yang for more than 20 years. JYYS granule contains* Bidens pilosa* Linn. (30 g),* Cornus officinalis* Sieb. et Zucc. (15 g),* Fallopia multiflora* (Thunb.) Harald. (12 g),* Scrophularia ningpoensis* Hemsl. (15 g),* Cyathula officinalis* Kuan (15 g), and* Alisma plantago-aquatica* Linn. (15 g). It has been reported that extracts from* Bidens pilosa and Cornus officinalis* can ameliorate hypertension and renal injury, respectively [24–26]. Extracts from* Fallopia multiflora* show antioxidant activity [27]. Extracts from* Cyathula officinalis* inhibit arterial remodeling in spontaneously hypertensive rats [28]. Components from* Alisma plantago-aquatica* show significant anti-chronic-prostatitis activity, anti-inflammatory, and antioxidant activities [29–31]. Finally, most studies about JYYS granule have been reported in Chinese, and JYYS granule had a therapeutic and protective effect on hypertension and early renal damage of hypertension by decreasing content of angiotensin II (AngII) and expression of TNF-*α* [32]. Consequently, clinic and experimental data have demonstrated that JYYS granule can attenuate hypertension-induced renal injury. However, the molecular mechanism of JYYS granule remains unknown in the treatment of hypertensive renal injury.

In this study, we determined the effects of JYYS granule on hypertensive renal damage and we explored the role of NF-*κ*B signaling in JYYS granule-mediated protection of the kidney. Our study will offer a theoretical basis for widespread use of JYYS granule in the management of hypertension and hypertensive nephropathy.

## 2. Methods and Materials

### 2.1. Preparation of Jiang Ya Yi Shen (JYYS) Granule

The JYYS granule was provided by Jiangsu Province Hospital of TCM (Nanjing, Jiangsu, China). The JYYS granule consists of* Bidens pilosa* Linn. (30 g),* Cornus officinalis* Sieb. et Zucc. (15 g),* Fallopia multiflora* (Thunb.) Harald. (12 g),* Scrophularia ningpoensis* Hemsl. (15 g),* Cyathula officinalis* Kuan (15 g), and* Alisma plantago-aquatica* Linn. (15 g). Each of these Chinese herbs was boiled with distilled water, and we got the extracts from the aqueous solution with ethanol. In the next step, the extracts were concentrated by freeze drying, which is the process of removing moisture from a frozen product in a vacuum. All the final crude products were mixed together and ground into powder. Finally, they were made into JYYS granule.

### 2.2. Collection of Clinical Patients

Patients were selected for this study according to the criteria of the World Health Organization (WHO). They were the patients in our hospital from the 2006th to 2009th year. They are 31 males and 9 females, and their ages are 57±5.12 (mean ± SD). The patients were collected and considered as having early hypertension-caused renal damage if they met these criteria: (1) the content of *β*2-MG in urine is equal to or greater than 0.227 mg/L; (2) the content of albumin in urine is equal to or greater than 13.4 mg/L; (3) the content of NAG in urine is equal to or greater than 19 U/L. The patients were excluded if they suffered diabetes and hypertension. They were randomly divided into the placebo and JYYS group, 20 patients for each group. Here, Color Doppler Ultrasonography was used to determine blood indicators in patients with hypertension-induced kidney injury in the early stage. We measured the content of NAG, Alb, and *β*2-MG in patients' urine after JYYS granule treatment. We also examined the effect of JYYS granule on the hemodynamic parameters of the renal artery in patients with hypertension-induced renal damage.

### 2.3. Spontaneously Hypertensive Rats (SHR) Animal Experiments

The Wistar-Kyoto (WKY) rats and SHR were purchased from the Academy of Military Medical Sciences, Beijing, China, and were housed in a standard vivarium with 12 h/12 h light/dark cycles, temperature of 23±1°C, and free access to food and water. The study was approved by the Animal Care and Protection Committee of Jiangsu Province Hospital of Traditional Chinese Medicine. All the experimental rats were kept under the above conditions for 4 days' acclimation. Here, the WKY rats were used as normal control and they were administrated with water (i.g.). A total of 40 SHR at the age of 12 weeks were randomly divided into 5 groups: (1) the placebo group (SHR): each SHR was administrated with 5 *μ*L of water per day (i.g.); (2) positive group (Benazepril Hydrochloride): each SHR was administrated with 3.33 mg/kg body weight per day (i.g.) and 100 g of this drug was diluted in 1 mL; (3) JYYS (H) group: each SHR was administered with 10 g/kg body weight per day and 100 g of JYYS granule was diluted in 1 mL (i.g.); (4) JYYS (M) group: each SHR was administered with 5 g/kg body weight per day and 100 g of JYYS granule was diluted in 1 mL (i.g.); (5) JYYS (L) group: each SHR was administered with 2.5 g/kg body weight per day and 100 g of JYYS granule was diluted in 1 mL (i.g.). After JYYS granule treatment for 8 weeks, arterial blood pressure was measured in conscious, restrained rats by a tail cuff system in a dark place at the temperature of 22°C in the morning. These rats were subjected to 15 acclimation measurements in a restraint holder and then blood pressure was calculated from the average of 10 measurement cycles. Meanwhile, their urine samples were collected in individual metabolic cages, and then their blood was collected by cardiac puncture after anesthesia, and the kidneys were collected for immunohistochemistry, western blot, and real-time polymerase chain reaction (PCR) as described [33].

### 2.4. Histology and Immunohistochemistry Staining (IHC)

The renal tissues were excised and fixed in 10% formalin buffer. The fixed specimens were embedded in paraffin blocks, sectioned, and stained with hematoxylin and eosin (H&E staining). These sections were observed under a Carl Zeiss microscope (Axio Observer A1, Jena, Germany). For electron microscopy investigations, kidney tissues were fixed with 2.5% glutaraldehyde for 3 hours, rinsed in phosphate buffer saline (PBS), and fixed in 1% OsO4, dehydrated, and embedded in EPON. The sections were contrasted with uranyl acetate and lead citrate and photographed in a JEM-2100 transmission electron microscope.

The kidney sections were subjected to dewaxing in xylene and dehydration in graded ethanol. 3% H_2_O_2_ was used to block the activity of endogenous peroxidase for 10 min. Then, the sections were heated to 100°C in 0.1 M citrate buffer (pH 6.0) for 30 min to retrieve the antigens. These renal tissues were incubated with the indicated antibodies at 4°C overnight. The tissues were incubated with second antibodies, which were horseradish peroxidase-conjugated anti-rabbit or anti-mouse IgG (Envision kit, Dako, Denmark) following the manufacturer's instructions. Finally, these sections were stained with hematoxylin. The stained sections were photographed with a Carl Zeiss microscope (Axio Observer A1, Jena, Germany). The semiquantitative analysis was based on optical density with Image J software by 2 investigators blinded to the experimental design [34].

### 2.5. Immunofluorescence

The kidney tissue was cut into 4 *μ*m frozen slices. After 10 min drying the glass sections were used for analysis. The embedding agent (OCT) in the biopsy was removed by washing with PBS and they were placed in a wet and dark box. These slices were blocked with a 100 *μ*L sealing solution at room temperature for 1 h. Then, the sections were incubated with the indicated primary antibodies at 4°C overnight. After washing with PBS for three time times per 5 min, the tissues were incubated with second antibodies, which were carrying with fluorescence (Thermo Fisher Scientific, Inc.) following the manufacturer's instructions. Finally, the image analysis system of a laser scanning confocal microscope was used to evaluate the expression of target genes in this study.

### 2.6. Enzyme-Linked Immunosorbent Assay (ELISA)

The effects of JYYS granule on the production of transforming growth factor-*β* (TGF-*β*) in the serum of patients or animals were measured with ELISA kits according to the manufacturer's instructions (R&D Systems, Inc., 614 McKinley Place NE, MN, USA).

### 2.7. Urine and Serum Biochemical Tests

Urine Alb and *β*2-MG were determined by biuret method and radioimmunity kits, respectively. Rats' urine and serum creatinine levels were measured using an automated analyzer according to the manufacturer's instructions.

### 2.8. Western Blot Analysis

After administration of JYYS granule to the animal model, the renal tissues were analyzed by immunoblotting. Total protein was prepared by gently lysing cells for 30 min with a lysis buffer (20 mM sucrose, 1 mM EDTA, 20 *μ*M Tris-Cl, pH 7.2, 1 mM DTT, 10 mM KCl, 1.5 mM MgCl_2_, 5 *μ*g/mL pepstatin A, 10 *μ*g/mL leupeptin, and 2 *μ*g/mL aprotinin). For western blot analysis, an equal amount of protein was subjected to electrophoresis on sodium dodecyl sulfate- (SDS-) polyacrylamide gels and the gel was transferred to a polyvinylidene fluoride (PVDF) membrane. Blots were incubated overnight at 4°C with the desired antibodies and then incubated with the diluted enzyme-linked secondary antibodies before visualizing by enhanced chemiluminescence according to the recommended procedure. The results are representative of three independent experiments. The enhanced chemiluminescence- (ECL-) detecting reagent was purchased from Pierce Biotechnology (Rockford, IL, USA). Antibodies against glyceraldehyde-3-phosphate dehydrogenase (GAPDH), TGF-*β*, collagen-IV, matrix metalloprotein (MMP-9), tissue inhibitor of metalloproteinase-1 (TIMP-1), RelA (p65), and I*κ*B were purchased from Cell Signaling Technology, Inc. (Danvers, MA, USA).

### 2.9. RNA Isolation and Real-Time PCR

Total RNA was isolated from renal tissues using TRIzol reagent (Thermo Fisher Scientific, Inc.). Then, cDNA synthesis was conducted using the SuperScript III RT kit (Thermo Fisher Scientific, Inc.). Real-time PCR reactions were carried out using the SYBR Green PCR Master Mix (Thermo Fisher Scientific, Inc.) in an ABI 7500 thermal cycler (Thermo Fisher Scientific, Inc.). GAPDH was used as an internal control. The primers (GenScript Co., Ltd., Nanjing, China) were listed below: GAPDH, 5′-CAC CAT CTT CCA GGA GCG AG-3′ (forward) and 5′-GCA GGA GGC ATT GCT GAT-3′(reverse); TNF-*α*, 5′-GCG ACG TGG AAC TGG CAG AAG-3′ (forward) and 5′-TCC ATG CCG TTG GCC AGG AGG-3′ (reverse); IL-6, 5′-TGG AGT CAC AGA AGG AGT GGC TA-3′ (forward) and 5′-TGA CCA CAG TGA GGA ATG TCC AC-3′ (reverse); TGF-*β*1, 5′-CTG TGG AGC AAC ACG TAG AAC TCT-3′ (forward) and 5′-TGT ATT CCG TCT CCT TGG TTC A-3′ (reverse); collagen (COL) IV, 5′-CCA GGC AAG GAC GGA AAA-3′ (forward) and 5′-GCC CAG AGT ACC AAG GTC T-3′ (reverse); COL I, 5′-GGC CAA GAA GAC ATC CCT GA-3′ (forward) and 5′-CGT GCC ATT GTG GCA GAT AC-3′ (reverse); fibronectin, 5′-TCG CTT TGA CTT CAC CAC CAG-3′ (forward) and 5′-CCT CGC TCA GTT CGT ACT CCA C-3′ (reverse); MMP-9, 5′-CGT GTC TGG AGA TTC GAC TTG A-3′ (forward) and 5′-TGG AAG ATC GTG TGA GTT CC-3′ (reverse); TIMP-1, 5′-TCT GGC ATC CTC TTG TTG CT-3′ (forward) and 5′-CAC AGC CAG CAC TAT AGG TCT T-3′ (reverse); ICAM-1, 5′-CCT GGG TCA TAA TTG TTG GTG-3′ (forward) and 5′-AGG AAG TCA GCC TTT CTT GG-3′ (reverse); MCP-1, 5′-GCT GCT ACT CAT TCA CTG GCA A-3′ (forward) and 5′-TGC TGC TGG TGA TTC TCT TGT A-3′ (reverse); p65, 5′-CAT ACG CTG ACC CTA GCC TG-3′ (forward) and 5′-TTT CTT CAA TCC GGT GGC GA-3′ (reverse); I*κ*B, 5′-CTC AAG AAG GAG CGG TTG GT-3′ (forward) and 5′-CCA AGT GCA GGA ACG AGT CT-3′ (reverse). The thermocycling conditions were as follows: initial denaturation of 94°C for 5 min, followed by 40 cycles of 94°C for 30 sec and 58°C for 30 sec, and final extension of 72°C for 15 sec. Each reaction was conducted in duplicate. Relative mRNA expression of genes was evaluated using the 2^−∆∆Cq^ method.

### 2.10. Statistical Analysis

All the data in this study are expressed as the mean ± standard deviation. Comparisons between two groups were performed using Student's* t*-test. Comparisons among multiple groups were performed with one-way analysis of variance followed by Tukey's honestly significant difference test. Statistical analyses were conducted using SPSS 17.0 software (SPSS, Inc., Chicago, IL, USA).* P*< 0.05 was considered to indicate a statistically significant difference.

## 3. Results

### 3.1. JYYS Granule Preserves Renal Function in SHR

**Figure 1:**
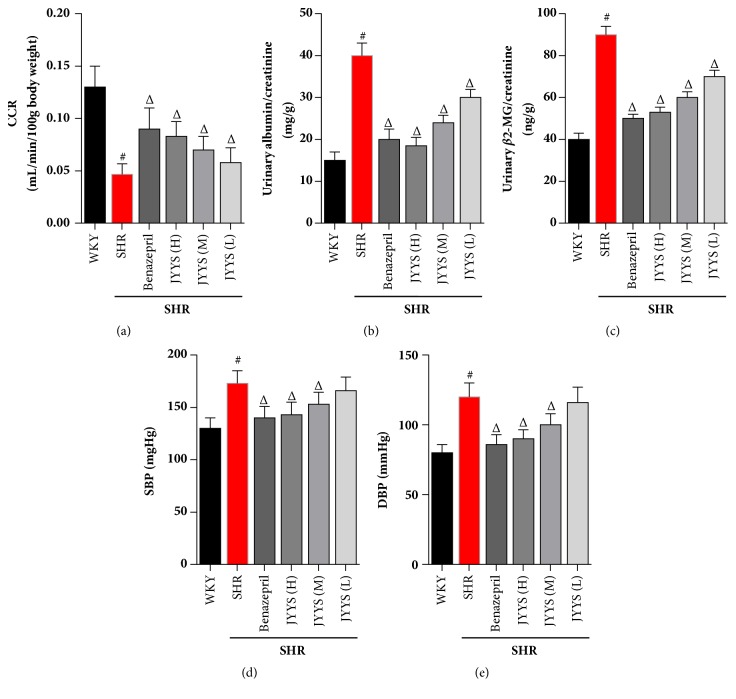
JYYS granule preserves renal function in SHR. Effects of JYYS granule on CCR, urinary albumin/creatinine, *β*2-MG/creatinine, and arteria caudalis pressure levels at the age of 24 weeks in SHR. (a) CCR. (b) Urinary albumin/creatinine. (c) Urinary *β*2-MG/creatinine. (d) SBP. (e) DBP. All data are expressed as mean ± SD, n=10, #*P* <0.05 versus WKY group, Δ<0.05 versus SHR group.

Firstly, we determined the effects of JYYS granule on indicators of renal function including CCR, urinary albumin/creatinine, *β*2-MG/creatinine, and arteria caudalis pressure in SHR (Figure 1). Compared with the WKY group, CCR was significantly decreased and the ratio of urinary albumin/creatinine and *β*2-MG/creatinine was increased in the SHR group (Figures 1(a) and 1(b)). In this study, we selected Benazepril as the positive group in SHR. After JYYS granule and Benazepril treatment, the decrease of CCR was significantly blunted, and the increase in the ratio of urinary albumin/creatinine and *β*2-MG/creatinine was reversed in the SHR rats (Figures 1(a), 1(b), and 1(c)). Additionally, we found that systolic blood pressure (SBP) and diastolic blood pressure (DBP) were obviously increased in SHR at the 12th week compared with the WKY group. Interestingly, the SBP and DBP in SHR were reduced by administration of Benazepril and JYYS granule (high concentration and median concentration) compared with SHR of placebo treatment (Figures 1(d) and 1(e)).

### 3.2. JYYS Granule Attenuates Renal Damage in SHR

**Figure 2:**
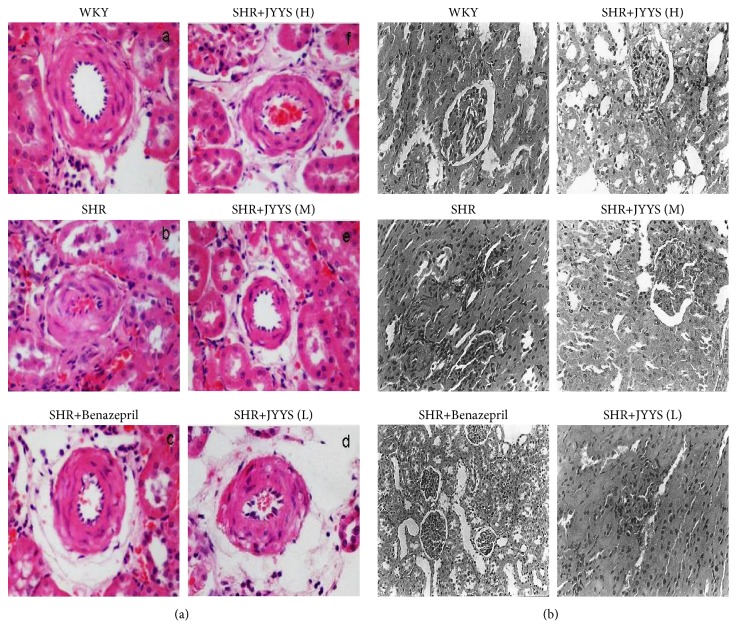
JYYS granule mitigates histological changes of glomerular injury in SHR. (a) HE staining under light microscopy (×200): WKY group, SHR group, Benazepril group (SHR), JYYS (H), JYYS (M), and JYYS (L). In the SHR group, glomeruli showed hyperemia, focal tubular epithelial cells were swollen with inflammatory cells infiltration, and partial arterioles showed wall thickening. These abnormal changes were attenuated by Benazepril as well as JYYS granule. No abnormality was found in the WKY group. (b) HE staining under electron microscopy (×4000): WKY group, SHR group, Benazepril group (SHR), JYYS (H), JYYS (M), and JYYS (L). In the SHR group, there was basal membrane thickening, and focal foot processes were fused and disarranged. No abnormality was found in the WKY group. The dysfunction was mitigated by Benazepril and JYYS granule.

Compared with the WKY group, the data showed that the renal arteriole and small arteries vessel walls presented hypertrophy in the SHR group, and the mesangial cells underwent mild hyperplasia, focal renal tubular epithelial cells became swollen, and inflammatory cell infiltrated by using light microscopic examinations (Figure 2(a)). After administration of JYYS granule and Benazepril, the mesangial region showed no hyperplasia and the renal capsule did not expand. Meanwhile, we did not observe abnormalities in the renal tubules, inflammatory cell infiltration, and small artery thickening (Figure 2(a)). Similarly, by using electron microscopic observations, we observed that the mesangial region in the SHR group underwent mild hyperplasia compared with the WKY group (Figure 2(b)). The basement membrane became thickened, and capillary loops were reduced and collapsed (Figure 2(b)). The abnormal changes in the kidney may be significantly ameliorated by JYYS granule treatment and Benazepril treatment (Figure 2(b)).

### 3.3. JYYS Granule Decreases Profibrogenic Genes and Extracellular Matrix Decomposition in Kidney of SHR

**Figure 3:**
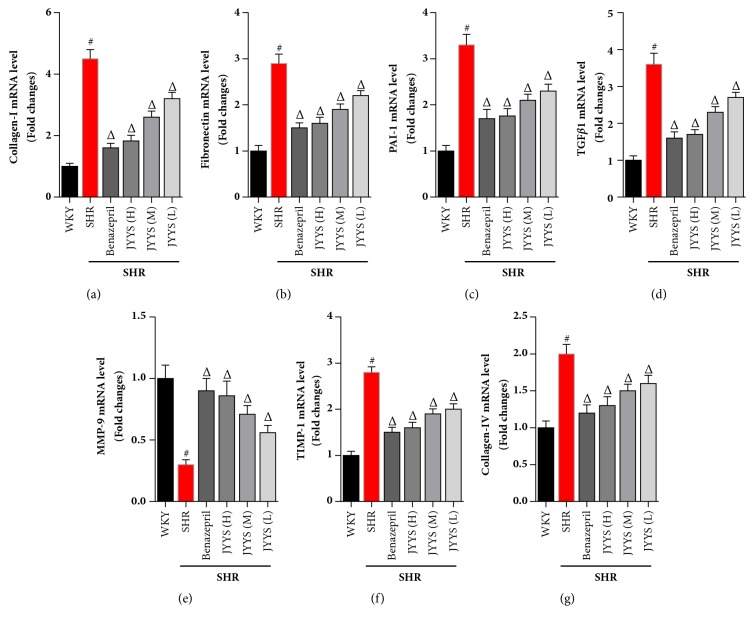
JYYS granule decreases mRNA levels of profibrogenic genes and extracellular matrix decomposition in the kidneys of SHR. Real-time PCR was performed to determine the effects of JYYS granule on renal fibrotic genes and extracellular matrix genes (n=10). (a) Collagen-I; (b) fibronectin; (c) PAI-1; (d) TGF-*β*1; (e) TIMP-1; (f) collagen-IV; (g) MMP-9. The values were normalized to the GAPDH values and then expressed as relative quantification. Data are expressed as mean ± SD, n=10, #*P* <0.05 versus WKY group, Δ<0.05 versus SHR group.

In this study, we firstly analyzed the mRNA expression of collagen-I, fibronectin, plasminogen activator inhibitor-1 (PAI-1), TGF-*β*1, TIMP-1, collagen-IV, and MMP-9 by conducting real-time PCR. The data showed that collagen-I, fibronectin, PAI-1, TGF-*β*1, TIMP-1, and collagen-IV mRNA level was much higher in SHR than in WKY rats (Figures 3(a), 3(b), 3(c), 3(d), 3(e), and 3(f)). MMP-9 was lower in SHR than in WKY rats (Figure 3(g)). However, JYYS granule and Benazepril can significantly downregulate collagen-I, fibronectin, PAI-1, TGF-*β*1, TIMP-1, and collagen-IV mRNA expression and upregulate MMP-9 mRNA expression (Figure 3).

**Figure 4:**
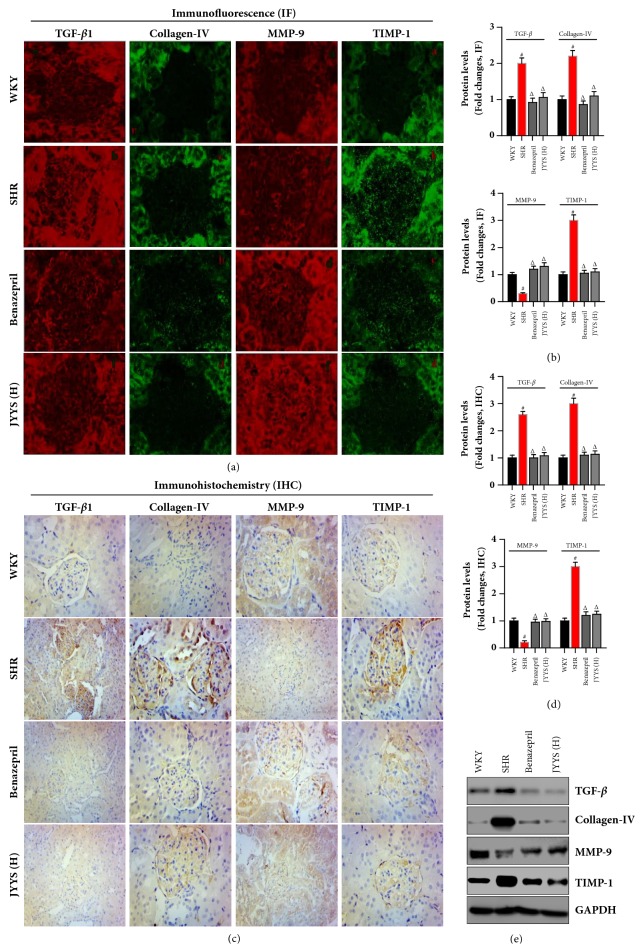
JYYS granule decreases protein levels of renal profibrogenic genes and extracellular matrix decomposition in SHR. ((a) and (b)) Immunofluorescence (IF) was carried out to examine the effects of JYYS granule on renal TGF-*β*1, collagen-IV, MMP-9, and TIMP-1 expression; and the semiquantification of these genes was based on the fluorescence intensity. Data are expressed as mean ± SD, n=10, #*P* <0.05 versus WKY group, Δ<0.05 versus SHR group. ((c) and (d)) Immunohistochemistry (IHC) was carried out to examine the effects of JYYS granule on renal TGF-*β*1, collagen-IV, MMP-9, and TIMP-1 expression and the semiquantification of these genes was based on the intensity. Data are expressed as mean ± SD, n=10, #*P* <0.05 versus WKY group, Δ<0.05 versus SHR group. (e) Western blot was carried out to examine the effects of JYYS granule on renal TGF-*β*1, collagen-IV, MMP-9, and TIMP-1 expression.

Secondly, we examined the protein expression of TGF-*β*1, collagen-IV, MMP-9, and TIMP-1 in the kidney by immunofluorescence, immunohistochemistry, and western blot (Figures 4(a), 4(c), and 4(e)). TGF-*β*1, collagen-IV, and TIMP-1 protein expression was remarkably elevated in SHR compared with WKY rats while the MMP-9 protein was reduced in SHR compared with WKY rats. Evidenced by immunofluorescence, JYYS granule and Benazepril treatment decreased the expression of TGF-*β*1, collagen-IV, and TIMP-1 protein and promoted the expression of MMP-9 in SHR renal tissue (Figures 4(a) and 4(b)). Similarly, these changes were also demonstrated by using immunohistochemistry and western blot analysis (Figures 4(c), 4(d), and 4(e)).

**Figure 5:**
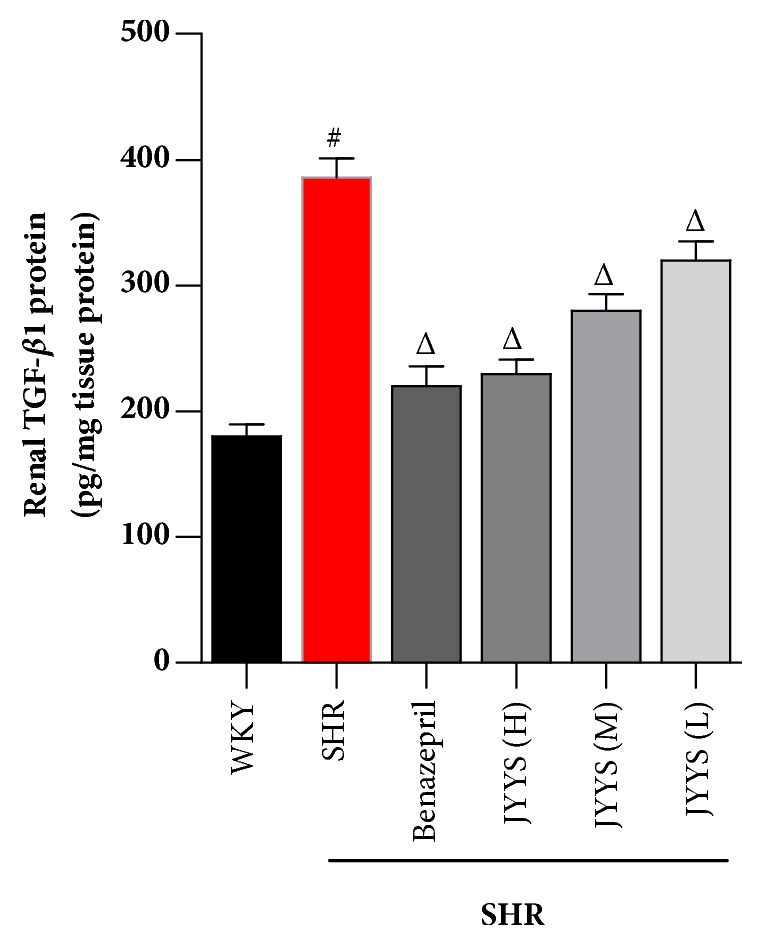
JYYS granule reduces renal cortical TGF-*β*1 protein level in SHR. ELISA was conducted to measure the renal cortical homogenate TGF-*β*1 protein levels of each group. Data are expressed as mean ± SD, n=10, #*P* <0.05 versus WKY group, Δ<0.05 versus SHR group.

Besides, for determining the effect of JYYS granule on TGF-*β*1 protein synthesis in SHR, renal tissue homogenate was measured using TGF-*β* ELISA kit. The TGF-*β* level was significantly higher in SHR than in WKY rats (*P*<0.05). There was a 50% reduction in TGF-*β* by Benazepril and a high dose of JYYS treatment in SHR (Figure 5).

### 3.4. JYYS Granule Ameliorates Microinflammatory and Renal Inflammation Genes in SHR

**Figure 6:**
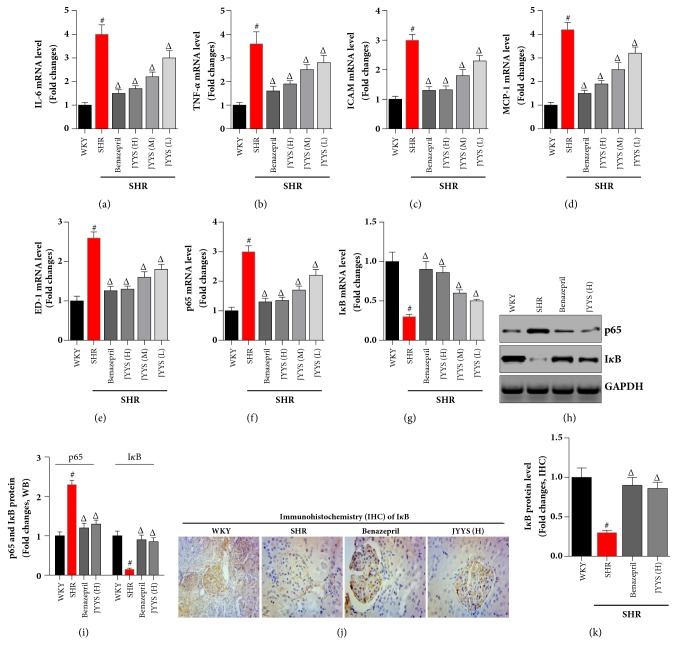
JYYS granule inhibited expression of microinflammatory genes and ameliorates NF-*κ*B mediated renal inflammation in SHR. (a~d) Real-time PCR was used to analyze the mRNA expression of microinflammatory genes, including IL-6 (a), TNF-*α* (b), ICAM-1 (c), and MCP-1 (d); results are showed as mean ± SD, n=10, #*P* <0.05 versus WKY group, Δ<0.05 versus SHR group. (e) Real-time PCR was conducted to analyze I*κ*B mRNA expression; data are expressed as mean ± SD, n=10, #*P* <0.05 versus WKY group, Δ<0.05 versus SHR group. ((f) and (g)) Immunohistochemistry (IHC) was performed to detect the effects of JYYS granule on renal I*κ*B protein expression and the semiquantification of these genes was based on the fluorescence intensity (f); data are expressed as mean ± SD, n=10, #*P* <0.05 versus WKY group, Δ<0.05 versus SHR group (g). ((h) and (i)) Western blot was conducted to evaluate the effects of JYYS granule on renal I*κ*B protein expression (h); data are expressed as mean ± SD, n=10, #*P* <0.05 versus WKY group, Δ<0.05 versus SHR group (i). ((j) and (k)) Immunohistochemistry (IHC) was carried out to examine the effects of JYYS granule on renal I*κ*B expression and the semiquantification of these genes was based on the intensity. Data are expressed as mean ± SD, n=10, #*P* <0.05 versus WKY group, Δ<0.05 versus SHR group.

Because macrophage-originated microinflammatory cytokines are essential and fundamental in the pathogenesis of hypertensive renal injury, they were examined by real-time PCR. The macrophage-associated microinflammatory cytokines IL-6, TNF-*α*, ICAM-1, and MCP-1 were expressed much higher in SHR than in the WKY group. Consistently, JYYS granule obviously downregulated expression of IL-6, TNF-*α*, ICAM, and MCP-1 in SHR (Figures 6(a), 6(b), 6(c), and 6(d)). Moreover, severe inflammation often appeared in both the glomeruli and interstitium of SHR. This pathological change was significantly attenuated by JYYS granule treatment. To confirm these findings and to avoid possible bias in tissue sectioning, selection, and counting, ectodermal dysplasia-1 (ED-1) protein expression was analyzed by western blot on whole-kidney homogenates. The data showed that ED-1 protein was downregulated by JYYS granule in a dose dependent manner (Figure 6(e)).

### 3.5. JYYS Granule Exhibits Its Inhibitory Effects of Hypertensive Renal Damage by Enhancing the I*κ*B Expression

To further explore the molecular mechanism of JYYS granule-mediated protection of the kidney, we study the role of NF-*κ*B signaling in hypertensive renal injury. Real-time PCR and western blot demonstrated that p65 expression was increased in the SHR kidney and JYYS granule treatment remarkably decreased p65 expression resulting in suppression of NF-*κ*B signaling (Figures 6(f), 6(h), and 6(i)). Then, real-time PCR, western blot, and immunohistochemistry showed that the NF-*κ*B signaling inhibitor I*κ*B expression was increased in SHR compared with WKY rats (Figures 6(g), 6(h), 6(i), 6(j), and 6(k)). However, JYYS administration may significantly reverse the decreased I*κ*B expression leading to suppression of NF-*κ*B activity in the setting of hypertensive renal injury (Figures 6(g), 6(h), 6(i), 6(j), and 6(k)).

### 3.6. The Beneficial Effect of JYYS Granule on Patients with Hypertensive Renal Injury

The protection effect of JYYS granule on patients with hypertensive renal injury in the early stage was evaluated by the hemodynamic parameters of the renal artery. In this study, Color Doppler Ultrasonography was used to determine blood indicators in patients with hypertension-induced kidney injury in the early stage. We firstly found that, compared with the pretherapy patients in the JYYS granule group, the NAG (*P* < 0.01), Alb (*P* < 0.05), and *β*2-MG (*P* < 0.01) content in patients' urine were downregulated after JYYS granule treatment (*P* < 0.05), and, compared with the pretreatment patients, the NAG was also significantly decreased in the placebo groups (*P* < 0.05) (Table 1). Importantly, after JYYS granule treatment, the NAG and *β*2-MG content in patients' urine were significantly downregulated compared with these indicators in the placebo group (*P* < 0.05) (Table 1). Secondly, we evaluated the effects of JYYS granule on the hemodynamic parameters of the renal artery in patients with hypertension-induced renal damage. In the JYYS granule treatment group, JYYS decreased RI (both left and right renal artery) while it increased Vm (left renal artery) and Vdmin (right renal artery) (*P* < 0.05) (Table 2). Besides, the other indicators were also improved by JYYS administration compared with those in the placebo treatment; however, the levels of these indicators were not significantly reduced after JYYS granule treatment, compared with the placebo treatment (Table 2).

## 4. Discussion

In this study, JYYS granule significantly restored CCR and it reduced urinary albumin and *β*2-MG excretion and inhibited expression of profibrogenic genes and extracellular matrix genes including renal collagen-I, fibronectin, PAI-1, TGF*β*1, TIMP-1, and collagen-IV. Then, JYYS granule ameliorated renal pathological changes. Moreover, this granule was able to blunt NF-*κ*B activity by upregulating I*κ*B expression in SHR renal tissue and it was also able to suppress the expression of renal microinflammatory cytokines including IL-6, TGF-*α*, ICAM-1, and MCP-1 in SHR. These data suggest that JYYS granule is a perfect therapeutic drug in hypertensive renal injury through modulating the NF-*κ*B activity and microinflammatory factors' levels.

The SHR is the most widely used animal model of human essential hypertension and hypertensive renal injury. Hypertensive kidney damage of SHR occurs gradually with increasing age [35–37]. This whole process in SHR is similar to human hypertensive nephropathy [35–37]. In this study, SHR increased level of urinary albumin and *β*2-MG excretion and it also upregulated expression of profibrogenic genes and extracellular matrix genes compared with the WKY rats. JYYS granule has been a therapeutic drug for hypertension and early hypertension-caused renal damage by downregulating AngII and TNF-*α* level [32]. Extracts from* Bidens pilosa, Cornus officinalis, Fallopia multiflora, Cyathula officinalis*, and* Alisma plantago-aquatica* may ameliorate hypertension and renal injury [24–26] and show antioxidant activity [27], inhibit arterial remodeling in spontaneously hypertensive rats [28], and exhibit significant anti-chronic-prostatitis and anti-inflammatory activities [29–31]. In this study, the data also demonstrates that JYYS granule, in a dose dependent manner, could significantly decrease urinary albumin and *β*2-MG excretion, and it ameliorates renal pathological changes and suppresses expression of profibrogenic genes and extracellular matrix genes including renal collagen-I, fibronectin, PAI-1, TGF*β*1, TIMP-1, and collagen-IV. JYYS granule treatment significantly alters the blood pressure of SHR. Thus, these results indicate JYYS granule is a beneficial drug for antifibrosis and antihypertensive renal injury.

Inflammation is a detrimental setting in the development of renal injury in various nephropathies, such as chronic kidney disease (CKD). Both inflammatory cells and renal resident cells contribute to inflammation in SKD [38, 39]. Inflammatory cytokines including TNF-*α* and MCP-1 take part in inflammatory responses of renal diseases including hypertensive renal damage [40], diabetic nephropathy [41], and ischemia/reperfusion injury [42]. IL-6, TNF-*α*, ICAM-1, and MCP-1 are multifunctional microinflammatory cytokines which are involved in immunoreaction and inflammation of hypertensive renal damage [1, 43–47]. In our study, JYYS granule significantly attenuates renal pathologic injury and renal fibrosis by downregulating the expression of IL-6, TNF-*α*, ICAM-1, and MCP-1, which were obviously higher in the SHR group than in WKY rats. These microinflammatory mediators are target genes of NF-*κ*B, which was activated in SHR. Meanwhile, I*κ*B expression was significantly suppressed in the kidneys of SHR, further suggesting that NF-*κ*B signaling was stimulated in the setting of hypertensive nephropathy. We found that JYYS granule could elevate the expression of I*κ*B resulting in inhibition of NF-*κ*B pathway as well as its downstream target genes.

In clinic, the protection effect of JYYS granule on patients with hypertensive renal injury in the early stage was evaluated by the hemodynamic parameters of the renal artery. Firstly, compared with the pretherapy patients in the JYYS granule group, the NAG, Alb, and *β*2-MG content in patients' urine were significantly reduced after JYYS granule administration. Furthermore, we found that, after JYYS granule treatment, the NAG and *β*2-MG content in patients' urine were significantly decreased compared with these indicators after the placebo administration in the placebo group. Then, to measure effects of JYYS granule on the hemodynamic parameters of the renal artery in patients with hypertension-induced renal damage, the data showed that, in the JYYS granule treatment group, PI, Vm (m/s), Vdmin (m/s), and Vsmax (m/s) were significantly reduced after JYYS granule treatment. Additionally, the levels of resistance index (RI) (both left and right renal artery) were obviously decreased, and Vm (left renal artery) and Vdmin (right renal artery) were obviously increased after JYYS granule treatment compared to these in the placebo group after the control treatment.

## 5. Conclusions

JYYS granule treatment significantly ameliorates renal injury in the SHR model. This traditional medicine exhibited its protective effects by inhibiting NF-*κ*B signaling-mediated microinflammation cytokines including IL-6, TNF-*α*, ICAM-1, and MCP-1 in SHR. Importantly, the clinic data showed that JYYS granule could significantly improve the symptoms of patients with hypertensive renal damage. Thus, JYYS granule is a promising prescription for patients with hypertension and hypertensive renal damage.

## Figures and Tables

**Table 1:** Effects of JYYS granule on NAG, Alb, and *β*2-MG content in patients with hypertension-induced renal damage.

**Indicators**	treatment group (n=20)		placebo group (n=20)	
	pre-therapy	post-treatment	pre-therapy	post-treatment
NAG (IU/L)	36.91±11.41	25.55±11.01^#@^	37.92±12.16	30.77±10.76^*∗*^
Alb (mg/L)	26.47±7.31	20.12±7.68^*∗*^	26.70±7.61	24.38±7.82
*β*2-MG	0.42±0.10	0.28±0.10^#@^	0.43±0.10	0.37±0.10

Abbreviation: NAG (N-acetyl-D-glucosamine), Alb (albumin), and *β*2-MG (*β*2-microglobulin). *∗P*<0.05, ^#^*P*<0.01 versus pre-therapy in treatment group and placebo group; ^@^*P*<0.05 versus post-treatment in placebo group.

**Table 2:** Effects of JYYS granule on the hemodynamic parameters of the renal artery in patients with hypertension-induced renal damage.

Indicators	Renal artery	treatment group (n=20)		placebo group (n=20)	
		pre-therapy	post-treatment	pre-therapy	post-treatment
RI	Left	0.77±0.18	0.61±0.10^#@^	0.78±0.17	0.70±0.14*∗*
	Right	0.77±0.21	0.60±0.09^#@^	0.77±0.20	0.69±0.15*∗*
PI	Left	1.24±0.18	0.98±0.12*∗*	1.24±0.18	1.12±0.12
	Right	1.26±0.24	1.02±0.13*∗*	1.23±0.20	1.10±0.18
Vm (m/s)	Left	16.27±9.15	28.62±6.19^#@^	17.53±9.75	20.74±7.58
	Right	17.15±8.95	26.27±6.64*∗*	16.84±9.27	21.47±7.23
Vdmin (m/s)	Left	9.67±3.73	14.27±4.12*∗*	10.12±3.46	12.73±4.68
	Right	10.04±4.62	15.93±4.87*∗*^@^	9.89±4.01	12.59±4.44
Vsmax (m/s)	Left	26.74±6.67	35.27±6.99*∗*	27.47±5.78	30.98±5.94
	Right	28.09±6.98	36.42±7.01*∗*	28.43±6.26	32.79±7.05
AT (s)	Left	0.11±0.03	0.06±0.02*∗*	0.12±0.04	0.08±0.03*∗*
	Right	0.12±0.03	0.07±0.03*∗*	0.13±0.04	0.08±0.04*∗*

Abbreviation: RI (resistive index), PI (pulse index), AT (acceleration time), Vsmax (peak velocity of the systolic wave), Vdmin (minimum velocity of the diastolic stage), and Vm (mean velocity of the arterial blood). *∗P*<0.05, ^#^*P*<0.01 versus pre-therapy in treatment group and placebo group; ^@^*P*<0.05 versus post-treatment in placebo group.

## Data Availability

The data used to support the findings of this study are available from the corresponding author upon request.
